# Prevalence and antimicrobial susceptibility of enterotoxigenic extra-intestinal *Bacteroides fragilis* among 13-year collection of isolates in Kuwait

**DOI:** 10.1186/s12866-020-1703-4

**Published:** 2020-01-15

**Authors:** Wafaa Jamal, Fatima Bibi Khodakhast, Ameerah AlAzmi, Jόzsef Sόki, Ghayda AlHashem, Vincent O. Rotimi

**Affiliations:** 1 Department of Microbiology, Faculty of Medicine, Kuwait University, P. O. Box 24923, 13110 Safat, Kuwait; 2 Institute of Clinical Microbiology, University of Szeged, Szeged, Hungary

**Keywords:** *Bacteroides fragilis*, Enterotoxin, Carbapenem-resistance, Kuwait

## Abstract

**Background:**

Some strains of *Bacteroides fragilis* species are associated with diarrhea as a result of enterotoxin production (bft or fragilysin). Fragilysin is activated by C11 protease (*fpn*) and together with C10 protease (*bfp*) play a significant role in its invasiveness. The objectives of this study were to investigate the proportion of clinical isolates from extra-intestinal sources that are toxin producers and characterize the genes mediating toxin production. Clinical isolates submitted to our reference laboratory over the last 13 years were screened for toxin production using PCR technique. All stool isolates were excluded. The isolates were tested for their susceptibility to 8 antimicrobial agents by E test. Carbapenem resistance gene *cfiA* was detected by PCR.

**Results:**

A total of 421 *B. fragilis* isolates were viable. Out of these, *bft* was detected in 210 (49.9%) isolates. Of the 210 *bft*-positive isolates, 171 (81.4%), 33 (15.7%) and 6 (2.8%) harbored *bft*-1, *bft*-2, and *bft*-3 genes, respectively. Twenty (9.5%) of the *bft*-positive strains originated from bloodstream infections. Twenty-five, 20 and 9 strains harbored *bfp*-1, *bfp*-2 and *bfp*-3 gene, respectively. Two, 3, 4 *bfp* isotypes were detected simultaneously in some of strains. The resistance rates against amoxicillin-clavulanic acid was 32%, clindamycin 62%, cefoxitin 26%, imipenem 11%, meropenem 17%, metronidazole 4%, piperacillin 61% and tigecycline 14%. A chromosomally located *cfiA* gene that encode metallo-β-lactamase was identified in only 34 isolates (16.2%).

**Conclusions:**

The prevalence of enterotoxin-producing *B. fragilis* was high among the extra-intestinal isolates. Metronidazole was the most active agent against all isolates. There was no statistically significance difference between resistance rates among *bft*-positive and *bft*-negative isolates except for clindamycin.

## Backgrounds

*Bacteroides* species are obligate anaerobic members of the normal microbiota of the human gut. They are the commonest anaerobic bacteria associated with clinical infections particularly those associated with infections of the mucous membranes and adjacent tissues [1]. *B. fragilis* is a type species of the *Bacteroides* genus whose numerical population is far less than the other *Bacteroides* spp., (e.g. *B. thetaiotaomicron*, *B. distasonis* and *B. vulgatus*), in the normal gut microbiota. However, paradoxically, it is the most common non-spore forming anaerobic bacteria found in routine clinical specimens, e.g. those obtained from deep intra-abdominal abscesses, suppurative skin and soft tissue infections, infections following intra-abdominal and vaginal post-hysterectomy surgeries [1]. Recent studies on gut microbiota have suggested that *B. fragilis* can be the dominant species associated/adherent to the colonic mucosa in healthy individuals [2] contrary to the old believe that its contribution to the anaerobic microbiota of the gut is minimal based on studies on fecal samples.

An important virulence factor of *B. fragilis* is an enterotoxin. Enterotoxin-producing *B. fragilis* (ETBF) strain was first isolated from the stool of neonatal lambs with diarrhea in 1984 [3]. ETBF was later found to cause diarrhea in humans and has been defined as one of the 6 possible causes of diarrhea in children aged 1–5 years [4]. It has also been isolated infrequently from stool specimens of symptomatic adult patients [5]. Although some reports have put the prevalence of extra-intestinal ETBF at 6.2–38% [6, 7], the prevalence of ETBF strains among *B. fragilis* isolates from diarrheagenic and extra-intestinal clinical samples in Kuwait is unknown. The enterotoxin produced by *B. fragilis* is also known as fragilysin. It is a zinc-dependent, non-lethal, heat labile metalloprotease of about 20 kDa [8] which acts by cleaving the E-cadherin protein of zonula adherens and tight junctions in the intestinal epithelial cells leading to rearrangement of the actin of the cytoskeleton of the epithelial cells [9, 10]. So far the gene mediating fragilysin (*bft*) has been well characterized into 4 isotypes, namely *bft*-1, *bft*-2, *bft*-3 and *bft*-4 [11]; the latter is mainly found in the Far East [7].

In addition, *B. fragilis* has another virulence factor, an endotoxin/lipopolysaccharide (LPS) with a demonstrable toxicity [12]. Once *B. fragilis* is exposed to antibiotics, it liberates endotoxin more than other *Bacteroides* species which may explain why this species is associated with clinical infections and higher mortality rate [13].

Some studies have demonstrated an association between increased prevalence of ETBF strains and inflammatory bowel diseases, such as Crohn’s disease and ulcerative colitis [14], while a possible pathogenic role in the etiology of colorectal cancer [15] and bacteremia [16, 17] has been suggested. Two cystein peptidase types with pathogenic role have recently been described: *bfp*1–4 genes encoding C10 peptidase and *fpn* gene encoding C11 peptidase (fragipain). Potential link between C10 peptidase and the pathogenesis of inflammatory bowel disease and sepsis has been documented and fragipain has been shown to activate *B. fragilis* enterotoxin [17, 18].

The susceptibility of *B. fragilis* to metronidazole and carbapenem has been excellent with few anecdotal reports of resistance emerging in the literature over the last 3 decades [19–21]. They have remained the drugs extensively used for the treatment of infections caused by this opportunistic pathogen. The emerging reports of *B. fragilis* isolates resistant to these drugs are causing increasing concern to the infectious diseases and clinical microbiology experts worldwide. The trend of resistance to these drugs by *B. fragilis* in our country, and elsewhere in the Gulf countries, is not clearly defined at molecular level.

This study was designed to investigate the prevalence of ETBF and the *bft* genes among *B. fragilis* isolates collected over 13 years in the Anaerobic Reference Laboratory and to determine the presence of genes mediating carbapenemase (*cfiA*) production among carbapenem-resistant *B. fragilis* isolates. It was also planned to investigate the prevalence of *bfp*1–4 and *fpn* genes in *bft*-positive and *bft*-negative strains.

## Results

### Bacterial isolates

A total of 421 *B. fragilis* isolates were collected from the following different sources: wound infections (WIs), lower respiratory tract infections (LRTIs), bloodstream infections (BSIs), biopsy specimens, urine, and bile. As shown in Table 1, out of the 421 isolates, 210 (49.9%) harbored the *bft* gene. The majority of the *bft*-positive isolates were from WIs, followed by LRTIs, BSIs and biopsy specimens (BS).

**Table 1:** Distribution of *B. fragilis bft* subtypes among different sources of infections

Sources	Number (%)			Total (%)
	*bft-1*	*bft-2*	*bft-3*	
Wound infection	105 (50)	16 (7.6)	5 (2.4)	126 (60)
Respiratory tract infection	30 (14.3)	11 (5.2)	0	41 (19.5)
Bloodstream infection	16 (7.6)	4 (1.9)	0	20 (9.5)
Biopsy	17 (8.1)	2 (1)	1 (0.5)	20 (9.5)
Urine	1 (0.5)	0	0	1 (0.5)
Bile	1 (0.5)	0	0	1 (0.5)
Unknown	1 (0.5)	0	0	1 (0.5)
Total	171 (81.4)	33 (15.7)	6 (2.9)	210 (100)

The ages ranged from 1 month to 94 years (mean, 52 years). Of the 210 patients, 137 (65.2%) were males and 73 (34.8%) were females. Out of 39 isolates derived from patients with BSIs, 20 (51.2%) were positive for *bft* gene. The presence of *bfp*1–4 (C 10 protease gene) and *fpn* (C11 protease gene) were investigated in the 210 *bft*-positive *B. fragilis* strains using PCR. As shown in Table 2, the distribution of C10 protease (*bfp*) genes was the following: 25 (11.9%) isolates harbored *bfp-*1 gene, 20 (9.5%) *bfp-*2 and 9 (4.2%) *bfp-*3 isotypes alone.

**Table 2:** Distribution of *bfp* genes among 210 *bft-*positive *B. fragilis* strains

*bfp* gene in *B. fragilis* strains	No (%)
*bfp*1- positive	25 (11.9)
*bfp*2-positive	20 (9.5)
*bfp*3*-*positive	9 (4.2)
*bfp*4 positive	0 (0)
*bfp*1 and *bfp*2-positive	59 (28.1)
*bfp*1 and *bfp*3-positive	8 (3.8)
*bfp*1 and *bfp*4-positive	1 (0.47)
*bfp*2 and *bfp3-*positive	3 (1.4)
*bfp*1, *bfp*2 and *bfp*3-positive	31 (14.7)
*bfp*1, *bfp*2 and *bfp*4-positive	2 (0.95)
*bfp*1*, bfp*2*, bfp*3 and *bfp*4-positive	6 (12.6)
*bfp* –negative	46 (21.9)

Of the 210, 59 (28.1%) strains carried both *bfp-*1 and *bfp*-2 simultaneously, and 31 (14.7%) strains were positive for *bfp-*1, *bfp*-2 and *bfp*-3. In all, 46 (21.9%) isolates did not carry any of the tested *bfp* genes.

As shown in Table 3, 170 (80%) *B. fragilis* were positive for *fpn* (C11 protease) gene among the 210 *bft-*positive strains, 34 (20%) of which were *cfiA-*positive.

**Table 3:** Distribution of *fpn* and *bfp*1–4 genes in the *cfiA*-positive and *cfiA*-negative *B. fragilis*

*B. fragilis* strains (total no)	*cfiA*-positive (no = 34)	*cfiA*-negative (no = 176)
*bfp*1- positive (25)	11	14
*bfp*2- positive (20)	1	19
*bfp*3- positive (9)	0	9
*bfp*4- positive (0)	0	0
*bfp*1 and *bfp*2-positive (60)	12	48
*bfp*1 and *bfp*3-positive (8)	0	8
*bfp*1 and *bfp*4-positive (1)	0	1
*bfp*2 and *bfp*3-positive (3)	0	3
*bfp*1, *bfp*2 and *bfp*3-positive (31)	6	25
*bfp*1, *bfp*2, *bfp*4-positive (2)	2	0
*bfp*1, *bfp*2, *bfp*3 and *bfp*3-positive (6)	1	5
*bft-*negative isolates (46)	1	45
*fpn*-positive strains (170)	34	136

### Antimicrobial susceptibility testing and distribution of resistance cfiA gene

MIC range, MIC_50_ MIC_90_ and percentage of resistance of the tested antibiotics are shown in Table 4.

**Table 4:** Antimicrobial susceptibility for 421 *B. fragilis* isolates

Antimicrobial agent (breakpoint in μg/ml)	Range	MIC_50_	MIC_90_	% of resistance
Amoxicillin-clavulanic acid (4)	0.125 - > 256	2	> 256	32
Clindamycin (2)	< 0.016 - > 256	> 256	> 256	62
Imipenem (4)	0.023 - > 32	0.25	6	11
Meropenem (4)	0.023 - > 32	0.5	> 32	17
Metronidazole (8)	0.023 - > 256	1	2	4
Piperacillin (16)	0.19 - > 256	> 256	> 256	61
Cefoxitin (16)	0.032 - > 256	8	> 256	26
Tigecycline (4)	0.094–32	1	8	14

A total of 261 (62%) and 257 (61.1%) were resistant to clindamycin and piperacillin, respectively. Amoxicillin-clavulanic acid and cefoxitin had unacceptable high MIC values: 135 (32.1%) and 110 (26.1%) were resistant, respectively. Resistance rates to metronidazole, tigecycline, imipenem and meropenem were 4, 14, 11, and 17%, respectively. When resistance rates among *bft-*positive and *bft*-negative strains were compared, only resistance to clindamycin and tigecycline were higher among *bft*-positive than the *bft*-negative strains but only resistance to clindamycin attained statistically significant level (*P* = 0.048: CI 18.1–23.21) (Table 5).

**Table 5:** Antimicrobial resistance among *bft-*positive (210) and *bft-*negative (211) *B. fragilis*

Antibiotic (breakpoint in μg/ml)	No (%) of resistant *bft*-positive *B*. *fragilis*			Total no (%) of resistant *bft*-positive *B. fragilis*	No (%) of resistant *bft*-negative *B. fragilis*	*P* value	Confidence interval (CI)
	*bft*-1	*bft*-2	*bft*-3				
Amoxicillin-clavulanic acid (4)	56 (26.7)	2 (1)	2 (1)	60 (28.8)	37 (34.6)	0.354230	[−15.52, 27.12]
Clindamycin (2)	119 (56.7)	20 (9.5)	3 (1.4)	142 (67.6)	120 (56.9)	0.048847	[− 1.81, 23.21]
Imipenem (4)	16 (7.6)	1 (0.5)	1 (0.5)	18 (8.6)	27 (12.8)	0.482089	[−18.50, 26.90]
Meropenem (4)	31 (14.8)	2 (1)	1 (0.5)	34 (16.2)	39 (18.5)	0.479721	[−17.83, 22.43]
Metronidazole (8)	10 (4.8)	2 (1)	0	12 (5.7)	6 (2.8)	0.182954	[− 28.21, 34.01]
Piperacillin (16)	102 (48.6)	15 (7.1)	4 (1.9)	121 (57.6)	136 (64.5)	0.157430	[− 5.81, 19.61]
Cefoxitin (16)	36 (17.1)	2 (1)	1 (0.5)	39 (18.6)	70 (33.2)	0.079984	[−3.85, 33.05]
Tigecycline (4)	29 (13.8)	5 (2.4)	3 (1.4)	37 (17.6)	21 (10)	0.345271	[−13.89, 29.09]

Further analysis showed that 72 (34.2%) of *bft*-positive isolates were multidrug resistant (MDR), that is non-susceptibility to at least one agent in 3 or more different antimicrobial categories [22]. Out of the 72 MDR isolates, 64 (88.9%) and 8 (11.1%) were *bft*-1 and *bft*-2 subtypes, respectively. None of the *bft*-3 positive strains was multidrug-resistant.

Thirty-four (8.1%) and 19 (4.5%) of 421 isolates were resistant to meropenem and imipenem, respectively. Mechanism of carbapenem resistance revealed that all the carbapenem resistant strains were positive for the *cfiA* gene. This equates with 16.2% of the 210 *bft*-positive isolates. The sources of these resistant isolates were WIs [23], biopsy samples [4], BSI [3] and LRTIs [3]. A *cfiA* gene was detected in one *B. fragilis* strain that showed susceptibility to both imipenem and meropenem with MIC of 2 and 4 μg/ml, respectively. This isolate was cultured from a patient with wound infection. In addition, one *B. fragilis* isolate was resistant to both imipenem and meropenem with MIC of 8 and > 32 μg/ml, respectively, but *cfiA*-negative.

## Discussion

*B. fragilis* can cause serious clinical infections thought to be related to the production of enterotoxin (*bft*), among other virulence factors. This toxin is activated by C11 (*fpn*) and C10 proteases (*bfp*) which help in the invasiveness of the organism. It has been shown that *bft*-positive *B. fragilis* are more invasive than *bft*-negative isolates in different types of infections and that blood culture isolates are more likely to carry *bft* enterotoxin gene than other isolates [24]. The prevalence of 49.9% for the enterotoxin producing-extra intestinal *B. fragilis* in our study is relatively high when compared with the figures of 14.4% reported in Poland [25], 18.6%, in Japan [16], 6.2–38% in USA [6, 7], and 13–25% in Hungary [11, 26]. Our data showed that the majority (81.4%) of the isolates contained *bft*-1 isotype compared with 15.7% of *bft*-2 and 2.9% *bft-*3 isotypes. It is pertinent to note that no *bft*-4 isotype strain was detected in this series. This order of prevalence of the isotypes is partially concordant with previous reports by Scotto d’Abusco et al.*,* [27], Sarvari et al., [11] and Kierzkowska et al., [25]. In their study, Sarvari and colleagues [11] from Hungary reported the prevalence of 10% for the *bft*-1 isotype compared with 3% *bft*-2 but, unlike our study, they did not detect *bft-*3 isotype. This difference in the distribution of *bft* gene may be related to the severity of illness, prior antimicrobial therapy, type of the diet and thus gut flora and the method used to detect the enterotoxin. In addition, more than half (51.2%) of the isolates from bloodstream infections in Kuwait were enterotoxin producers which was much higher than previous reports from the USA and Japan (19–28.1% by Claros et al., 2006 and Kato et al, 1996, respectively) [16, 28]. In our study, simultaneous harboring of 2, 3 and 4 *bfp* isotypes occurred in 71, 33 and 6 isolates, respectively, which is higher than those reported by Sarvari et al., and among the most *bft*-positive strains, *bfp*-1 was the most prevalent isotype. This was discordant with the results of the study reported by Sarvari et al., in which the most common isotype was *bfp*-2 [11]. In addition, they did not find 4 isotypes in the same isolates [11]. Almost half of our isolates were *bft-*negative although they were pathogenic in a number of clinical scenarios. The explanation for this may be due to production of other virulence factors e.g. lipopolysaccharide (LPS) endotoxin especially after exposure to antibiotics [13] or both LPS and capsule that act as adhesion allowing the organism to become established at the site of infection and providing a nidus for abscess formation [1, 24].

The majority (60%) of *bft*-positive *B. fragilis* isolates were from wound infections which was higher than that reported from Germany and USA (10%; 24) and from Hungary (51%; 11), but lower than that reported in Warsaw, Poland (67.5%; 28). This probably implies that *bft*-positive strains are more pathogenic in wound infections than the *bft-*negative strains.

Antimicrobial resistance is a growing problem all over the world including resistance phenomena by *B. fragilis.* In this study, resistance to clindamycin (62%) was at an unacceptable level. This shows that *B. fragilis* in Kuwait are much more highly resistant to this agent than discordant reports elsewhere, such as 28.5% in Europe [29], 48.9% in Taiwan [30], 29.9% in USA [31] and 36.6% in China [32]. It is conceivable that the over use and abuse of this agent in almost all government and private hospitals as well as dental clinics in Kuwait is responsible for the alarming high resistance rate. Other relatively high unacceptable resistance level of 26% was recorded against cefoxitin. This is very disturbing finding as this agent is massively used for surgical prophylaxis by most of our surgeons in the country. Its empirical use must therefore be called to question. Metronidazole was the most active non-β-lactam drug with 4% resistance rate. Despite being an active agent in our country, this resistance rate is much higher than those reported from other countries around the world [29–32]. Although the resistance rate to amoxicillin-clavulanic acid in our study is marginally higher than those reported around the globe, it is nonetheless at a very uncomfortable high level (32%). Another very interesting but disturbing finding, in our study, is the relatively high tigecycline resistance level of 14%. This is too high when compared with findings reported in the European study (1.8%) by Nagy et al., [29], 0% in Taiwan study [30] and 5.4% in USA study [31].

*B. fragilis* resistance to carbapenem is often associated with production of a class B metallo-β- lactamase encoded by the chromosomal *cfiA* gene, in addition to outer membrane permeability barrier mechanism [33]. In our study, 11 and 17% of *B. fragilis* were resistant to imipenem and meropenem, respectively which are much higher than those reported in Europe (1.2% for imipenem) [29], USA (1.1% for imipenem and 2.5% meropenem) [31], Taiwan (8.5% imipenem and 9.9% meropenem) [30]. However, the *B. fragilis* resistance to imipenem and meropenem in our study were lower than reported in China (22.7 and 18.2%, respectively) [32]. One of our *B. fragilis* isolate was resistant to both imipenem and meropenem in the absence of *cfiA* gene which was similar to the report in studies by Soki et al., [34]. Our speculation is that this may be due to other mechanism of resistance, perhaps out membrane permeability problem. Detection of 16.2% *cfiA* gene is higher than 1.8% which was reported in Poland [25] but lower than that of 36.4% reported in China [32]. A *cfiA* gene was detected in one *B. fragilis* isolate that showed susceptibility to both imipenem and meropenem. This has been reported previously [35, 36] and can be explained by the absence of insertion sequence upstream the gene leading to poor expression of *cfiA* gene.

Limitation of the study include retrospective collection of isolates and clinical data. Response to therapy could not be determined and insertion sequence elements were not done in the *cfiA*-positive isolates. Although to our knowledge the most common mechanism of carbapenem resistance in *B. fragilis* is the production of cfiA metallo-β-lactamase via activation of the cfiA gene by IS elements (high level resistance) or by activation of its putative own promoter other possible mechanisms, such as other carbapenemase genes and AmpC gene, were not investigated.

## Conclusions

The prevalence of enterotoxin-producing *B. fragilis* strains among the clinical isolates of extra-intestinal origin was very high in our study. There was no statistically significance difference in the antibiotic resistance rates among *bft*-positive and *bft*-negative isolates except for clindamycin. In this study, metronidazole was the most active antimicrobial agent against enterotoxigenic *B. fragilis* isolates.

## Methods

### Study design

This was a multicenter prospective investigational study of stored *B. fragilis* isolates from 6 hospital microbiological laboratories (Mubarak, Amiri, Al Babtain, Ibn Sina, Adan and Maternity hospitals) in Kuwait. All clinical isolates obtained from proven cases of infections stored at − 80 °C were resuscitated and viable strains investigated for enterotoxin production.

### Bacterial strains

The bacterial strains were isolates collected during a 13-years period, from 2006 through 2018, and stored in the Anaerobe Reference Laboratory, Faculty of Medicine, Kuwait University. The viable *B. fragilis* isolates were from proven cases of intra-abdominal infections, lower respiratory tract infections, bloodstream infections, wound infections and abscesses, managed in the 6 hospitals. All isolates were stored, in Brain Heart Infusion (BHI, Oxoid limited, Basingstoke, Hampshire, UK) broth containing 20% glycerol, at -80 °C. During our investigation, isolates were subcultured on Brucella blood agar (Becton Dickinson, Heidelberg, Germany) incubated for 48 h at 37 °C, in an Anoxomat Anaerobic WS800 system™ (MART Microbiology BV, Lichtenvoorde, Netherlands), in an atmospheric condition of 85% N_2_, 10% CO_2_, 5% H_2_. The identification was confirmed by a Matrix Assisted Laser Desorption/Ionization-Time of Flight Mass Spectrometry (MALDI-TOF MS; bioMérieux, L’Etoile, Marcy, France) analysis.

### Antimicrobial susceptibility testing (AST)

The susceptibility of the isolates to 8 anti-anaerobic antibiotics was investigated by determining the minimum inhibitory concentrations (MICs) of the antibiotics using the E test method (bioMérieux) according to manufacturer’s instructions. The antibiotics tested were the following: amoxicillin-clavulanic acid, clindamycin, cefoxitin, imipenem, meropenem, metronidazole, piperacillin and tigecycline. Susceptibility profiles of the isolates were determined according to the interpretative criteria recommended by the CLSI, 2018 [37]. *B. fragilis* ATCC 25285, and *B. thetaiotaomicron* ATCC 29741 were included as control in each run. Results for the isolates were accepted if the quality control strains results were within the established CLSI ranges (CLSI, 2018). MIC_50_, MIC_90_ and percentage of resistance were calculated.

### Molecular detection of fragilysin (bft) gene

Using previously published procedure, a PCR was performed for the detection of *bft* gene in all the *B. fragilis* isolates using bftF and bftR primers [18]. The genes, primer sequences, cycling conditions are shown in Table 6. The following positive controls were used: *B. fragilis* R19811 (*bft*-1), *B. fragilis* 1 ATCC 43858 *(bft-*2), and *B. fragilis* GAI 96462 (*bft*-3). Sequencing of the amplicons of the internal fragments of *bft-*1*, bft-*2 and *bft-*3 were performed using a GenAmp PCR system 9700 by cycling sequencing with BigDye® Terminator (AB Applied Biosystems, Carlsbad, California, USA).

**Table 6:** The genes, primer sequence, cycling condition and reference for fragilysin *bft* gene, *fpn, bfp*1–4 genes and *cfiA* carbapenemase gene

Gene	Primer sequence (5′ to 3′)	Cycling condition	Product size (bp)	Reference
*bft*	bftF: CGAACTCGGTTTATGCAGTT bftR: GGATACATCAGCTGGGTTGT	95 °C/5 min; followed by 35 cycles of 95 °C/45 s, 56 °C /1 min, 72 °C/45 s, 72 °C/ 7 min	295	[18]
*fpn*	C11_protease_F: ATTCGGCCGATGCAAATGTG C11_protease_R: CGGAATCTCGGTAGGGAAC	95 °C 5 min; followed by 35 cycles of 95 °C/45 s; 56 °C /1 min; 72 °C/45 s, 72 °C/7 min	290	[11]
*bfp1*	C10_protease_F1: GCGGTGAACAAAGAACGACA C10_protease_R1: TCGCCTGAGCAACTGCAATA	95 °C 10 min; 35 cycles of 95 °C/15 s; 59 °C/30s; 72 °C/47 s; 72 °C/7 min	153	[11]
*bfp2*	C10_protease_F2: CGTACCAATTGCAATTGCGC C10_protease_R2: AGCTCCCGTGGCTTTATCTT	95 °C 10 min; 35 cycles of 95 °C/15 s; 59 °C/30 s; 72 °C/47 s; 72 °C/7 min	178	[11]
*bfp3*	C10_protease_F3: TTTGGAGTAGCAGCAGCAGA C10_protease_R3: TTTCTGGTTTCGGGTGTTTC	95 °C 10 min; 35 cycles of 95 °C/15 s; 59 °C/30 s; 72 °C/47 s; 72 °C/7 min	194	[11]
*bfp4*	C10_protease_F4: TACAACGGTGTTGGTGCAAG C10_protease_R4: ACACAAATGCGCCACTTCAT	95 °C 10 min; 35 cycles of 95 °C/15 s; 59 °C/30s; 72 °C/47 s; 72 °C/7 min	126	[11]
*cfiA*	cfiF: AATCGAAGGATGGGGTATGG cfiR: CGGTCAGTGAATCGGTGAAT	95 °C/10 min, followed by 35 cycles of 95 °C/15 s, 59 °C/30s, 72 °C/ 7 s; 72 °C/7 min	302	[38]

### Molecular detection of cfiA carbapenemase–producing gene

Production of carbapenemase by the isolates was detected in selected number of strains with very high MIC values for meropenem/imipenem using modified Hodge test. All *bft*-positive strains as well as imipenem and/or meropenem resistant *B. fragilis* (i.e. MIC ≥4 μg/ml) were screened for the presence of *cfi*A gene and confirmed by PCR, using published primers [38]. The genes, primer sequences, cycling conditions are given in Table 6. PCR was carried out in a volume of 25 μl. The PCR mix was obtained from Qiagen (Hilden, Germany) and the supernatant of boiled bacterial cells was used as a source of DNA template and the concentration of each primer was 25 pmol. PCR products was separated by agarose gel electrophoresis and stained with 1% ethidium bromide (Bio-Rad, Hercules, CA, USA) and visualized by UV light.

### Molecular analysis of C10 protease (bfp1–4) and C11 protease (fragipain, fpn) genes

For all *bft-*positive isolates, *bfp*1–4 and *fpn* genes in the C10 and C11 proteases, respectively, were investigated. They were investigated by PCR using the genes, primer sequences, cycling conditions given in Table 6 and the following control strains: *B. fragilis* 638R (*bfp1–4*) and *B. fragilis* ATCC 43859 (*fpn*) were included [17, 23, 28].

### Statistical evaluation

The EpiCalc 2000, version 1.02 (Brixton Heath, Llanidloes, Powys, Wales, UK) was used to compare two proportions-percentages with 95% confidence interval and one sided *P*-value.

## Data Availability

All data generated or analyzed during this study are publicly available and included in this published article.

## Abbreviations

AST: Antimicrobial susceptibility testing
*B. distasonis*: *Bacteroides distasonis*
*B. fragilis*: *Bacteroides fragilis*
*B. thetaiotaomicron*: *Bacteroides thetaiotaomicron*
*B. vulgatus*: *Bacteroides vulgatus*
*bfp*: C10 protease
*bft*: fragilysin enterotoxin
BHI: Brain Heart Infusion
BS: Biopsy specimens
BSIs: Bloodstream infections
*cfiA*: carbapenem resistance gene
CI: Confidence interval
CLSI: Clinical and Laboratory Standards Institute
CO_2_: Carbon dioxide
DNA: Deoxyribonucleotide
ETBF: Enterotoxin-producing *Bacteroides fragilis*
*fpn*: C11 protease
H_2_: Hydrogen
kDa: KiloDalton
LPS: Lipopoltsaccharide
LRTI: Lower respiratory tract infections
MALDI-TOF MS: Matrix Assisted Laser Desorption/Ionization-Time of Flight Mass Spectrometry
MDR: Multidrug resistant
MIC: Minimum inhibitory concentrations
MIC_50_: Minimum inhibitory concentrations that inhibited 90% of the isolates
MIC_90_: Minimum inhibitory concentrations that inhibited 90% of the isolates
N_2_: Nitrogen
PCR: Polymerase chain reaction
WIs: Wound infections
